# Bibliographic Review of Smith-Magenis Syndrome and its Psychopharmacological Management with Lithium: About a case

**DOI:** 10.1192/j.eurpsy.2025.1853

**Published:** 2025-08-26

**Authors:** M. Navarro Bardon, K. M. Rodríguez

**Affiliations:** 1 Psychiatry, SERGAS, Vigo, Spain

## Abstract

**Introduction:**

**Smith-Magenis Syndrome** (SMS) is a neurogenetic disorder caused by deletions on chromosome 17p11.2 or mutations in the **RAI1** gene. It is characterized by **intellectual disability**, **behavioral disturbances** like **aggression**, **impulsivity**, **self-injury **, and **sleep disruptions**. A hallmark feature of SMS is **inverted melatonin production**, leading to **daytime sleepiness** and **nighttime insomnia**, which exacerbate behaviors. Traditional treatments, such as **antipsychotics** and **SSRIs**, often show limited effectiveness and can cause side effects, including **metabolic syndrome**, **sedation**, and **extrapyramidal symptoms**.

**Lithium** has emerged as a promising alternative to manage **treatment-resistant behaviors** in SMS. Known for its **mood-stabilizing** properties in **bipolar disorder**, lithium modulates **dopamine** and **serotonin**, reduces **aggression**, and promotes **neuronal plasticity**. However, lithium requires **close monitoring** due to the risks of **nephrotoxicity**, **thyroid dysfunction**, and its **narrow therapeutic index**.

**Objectives:**

This study explores **lithium’s role** in managing **severe behavioral disturbances** in SMS, especially in patients unresponsive to conventional treatments. The objectives are: (1) to review the **literature** on lithium’s efficacy and safety in SMS and similar neurodevelopmental disorders, and (2) to present a **clinical case** of a 25-year-old SMS patient treated successfully with lithium after antipsychotics and SSRIs failed.

**Methods:**

A **literature review** was conducted using **PubMed** and **Web of Science**, focusing on articles published between 2013 and 2023 on lithium in SMS and related disorders. Additionally, the **clinical case** of a 25-year-old male with SMS, exhibiting **aggression** and **self-injury **, was documented. After other treatments failed, lithium was introduced with regular monitoring of **serum levels**, **renal**, and **thyroid function** throughout six months.

**Results:**

Literature supports lithium’s **efficacy** in reducing **aggression** and **impulsivity** in SMS. Lithium modulates **dopaminergic** and **serotonergic systems**, stabilizing mood and reducing disruptive behaviors. In the clinical case, the patient improved within two weeks of lithium therapy. Over six months, **aggression** and **self-injury** diminished significantly, with no adverse effects and stable **renal** and **thyroid function**.

**Conclusions:**

Lithium is an effective option for SMS patients with **treatment-resistant behavioral disturbances**, particularly **aggression** and **self-injury **. It offers a valuable alternative to antipsychotics and SSRIs, enhancing **emotional stability** and **quality of life**. However, careful **monitoring** is required to prevent toxicity. Further research is needed to confirm lithium’s long-term safety and efficacy in SMS.

**Disclosure of Interest:**

None Declared

